# The Interleukin-7 Receptor Signaling Pathway and Its Perturbation in Immunodeficiency, Autoimmune Disease and Lymphoid Malignancy

**DOI:** 10.3390/biom16020219

**Published:** 2026-02-02

**Authors:** Alister C. Ward

**Affiliations:** 1 School of Medicine, Deakin University, Geelong, VIC 3216, Australia; alister.ward@deakin.edu.au; 2 Institute for Mental and Physical Health and Clinical Translation, Deakin University, Geelong, VIC 3216, Australia

**Keywords:** autoimmunity, cytokine, immunodeficiency, IL-7, leukemia, JAK, STAT

## Abstract

Cell–cell signaling through a network of cytokine receptors is pivotal for normal immune development and function, with disruptions of these signaling pathways being implicated in a variety of immune cell diseases. Signaling via the interleukin-7 receptor (IL-7R) facilitates the development and homeostasis of various T-cell, B-cell and other immune-cell populations. This is reflected in the raft of mutants and variants of IL-7R components and downstream signaling molecules that have been identified in the context of immunodeficiencies, autoimmune disorders and lymphoid malignancies, but also through the use of pathway modulators as therapeutics. This review provides an overview of IL-7R biology, the role of mutations and variants affecting IL-7R signaling pathway components in the etiology of immune cell diseases and the specific therapies related to this pathway.

## 1. Introduction

A complex web of cytokines underpins normal immune development and function via binding to specific receptors expressed on the surface of target cells [1]. This causes the activation of associated Janus kinase (JAK) proteins that trigger intracellular signaling pathways involving signal transducer and activator of transcription (STAT) proteins and other signaling molecules. Collectively these pathways impact critical cellular outcomes such as commitment and differentiation along specific lineage trajectories and proliferation and survival, as well as mediating key functional aspects [2,3]. Signaling by the cytokine interleukin-7 (IL-7) through the IL-7 receptor (IL-7R) complex is critical for the development and function of several lymphoid cell populations, but is also perturbed in a range of immune diseases [4,5]. This review draws on relevant in vitro, animal and human studies to describe the IL-7R signaling pathway and its role in normal biology, the mutations and other variants that contribute to disease and how this pathway is harnessed therapeutically.

## 2. IL-7R Signaling and Its Role in Immune Cell Development and Function

**Figure 1:**
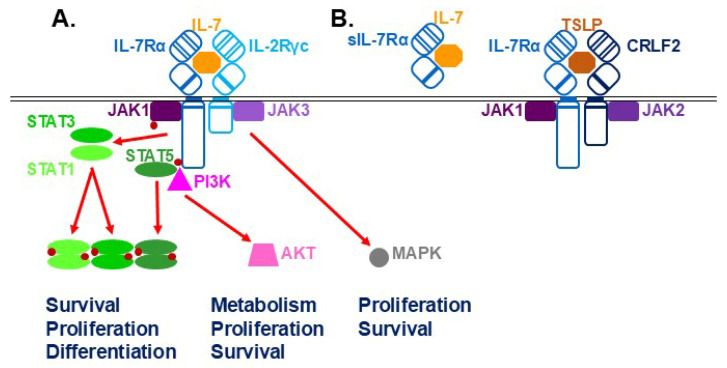
IL-7Rα and its signaling modalities. (**A**) IL-7 (orange) binds to the IL-7R complex, comprising IL-7Rα (dark blue) and IL-2Rγc (light blue). This leads to activation of the associated JAK1 (dark purple) and JAK3 (light purple) that phosphorylate tyrosine residues (red dots) on each other and on the receptor. STAT proteins are recruited (STAT5 via the IL-7Rα chain and both STAT1 and STAT3 via an alternate mechanism) and are, in turn, tyrosine-phosphorylated and dissociate from the receptor to form dimers that translocate to the nucleus, where they bind to specific DNA motifs to stimulate transcription of target genes. The p85 component of PI3K also docks to the IL-7Rα chain to stimulate AKT and downstream effector molecules, with the MAPK pathway being separately activated. (**B**) Other IL-7Rα modalities, including a soluble (s) form that binds IL-7, and an alternative complex with CRLF2, which is the receptor for TSLP.

Human IL-7 is a 152-residue glycosylated polypeptide that folds into a four α-helical bundle with bridging disulfide bonds typical of the class 1 family of cytokines [5]. IL-7 is produced in a variety of tissues, with the highest levels being found in the thymus and lymph nodes, which includes both immune cells and the stromal thymic epithelial cells (TECs) and fibroblast reticular cells (FRCs), and lesser levels in the bone marrow and spleen, but it is also observed in the intestine, liver and skin [6]. It binds to preformed IL-7R complexes (Figure 1A) consisting of a ligand-specific IL-7Rα chain (also called CD127) and the shared interleukin-2 receptor gamma common (IL-2Rγc, or simply γc) chain (CD132) [1,7]. The human IL-7Rα chain consists of a 219-residue extracellular region containing a cytokine receptor homology domain (CHD) comprising a pair of fibronectin type III (FNIII)-type modules connected by a short hinge. The N-terminal FNIII module contains structurally important paired cysteines, while the C-terminal module includes a conserved WSxWS motif important for receptor maturation. This is followed by a 25-residue transmembrane region and a 195-residue intracellular region including a Box 1 motif that binds JAK1 [4]. The IL-2Rγc chain has a similar overall structure, but with a shorter intracellular domain that binds JAK3 instead. IL-7 interacts with the CHD hinge region of the IL-7Rα chain, with the IL-2Rγc chain serving to enhance binding affinity and stability [8]. IL-7 binding causes rotation of the receptor chains, including their intracellular regions, which brings the associated JAK1 and JAK3 proteins into close proximity. This facilitates cross-phosphorylation, which enhances their activity, leading to further phosphorylation, including that of Y449 on the human IL-7Rα chain [7].

Various STAT proteins are recruited to the activated IL-7R complex: STAT5 via pY449 on IL-7Rα as well as STAT1 and STAT3 via alternative mechanisms [9,10]. The individual STATs are themselves tyrosine phosphorylated enabling dimer formation and nuclear translocation where they bind to regulatory sequences to influence transcription of key genes. STAT5 has been shown to exert a range of impacts, such as enhancing survival through impacting expression of BCL family members [11] and promoting proliferation via increased cyclin expression and p27kip destabilization [12], as well as participating in key aspects of differentiation [13], such as facilitating immunoglobulin (Ig) gene rearrangements in B cells [14]. The p85 subunit of phosphatidylinositide 3-kinase (PI3K) is also recruited, which leads to activation of PI3K that stimulates AKT (as well as the downstream GSK3, mTOR and FOXO) to promote proliferation, survival and metabolic changes [15]. The MAPK pathway can also be separately activated to impact cell phenotypes [16]. Receptor signaling is controlled by a number of mechanisms. These include the negative-feedback regulators SOCS1 and SOCS3, which are induced by IL-7 and act via direct inhibition of JAK activity and proteasomal degradation of receptor signaling components [17], and the activity of a variety of phosphatases [18], as well as clathrin-mediated internalization of the receptor complex [19]. There is also pathway crosstalk. For example, STAT5-mediated transcriptional responses can promote AKT activation to modulate glucose metabolism, which contributes to T cell survival [20]. Conversely, p85 competes with STAT5 at the pY449 docking site on the IL-7Rα to modulate T cell development and homeostasis [21].

The picture of IL-7R signaling is complicated by other factors (Figure 1B). Firstly, a soluble form of the IL-7Rα chain (sIL-7Rα) is generated through alternative splicing and serves to increase IL-7 half-life in circulation [22]. Secondly, there is sharing of receptor components. Thus, the IL-7Rα chain forms a separate complex with cytokine receptor-like factor 2 (CRLF2), which serves as the receptor for another cytokine thymic stomal lymphopoietin (TSLP) that mediates both distinct and overlapping roles [23]. In addition, both IL-2Rγc and the associated JAK3 are shared in the IL-2R, IL-4R, IL-9R, IL-15R and IL-21R complexes, which contain unique ligand-specific chains engaged with JAK1 and also impact immune cell development and function [1,24]. Indeed, IL-7Rα has been shown to sequester IL-2Rγc to limit signaling via IL-2, IL-4 and IL-21 due to its higher affinity for this receptor chain [25,26]. Conversely, IL-2 signaling can reduce IL-7Rα expression to dampen IL-7 responsiveness [27], while IL-2 and IL-7 can also induce the expression of the other’s ligand-specific receptor chain to optimize cellular responses [28]. In a similar way, co-operation has been demonstrated between TSLP and IL-7 [23] and between IL-7 and T cell receptor (TCR) engagement [29].

IL-7R signaling makes a broad contribution to the development and homeostasis of a variety of immune cell populations [30]. Much of the specificity is determined by the expression pattern of the IL-7Rα chain, which includes common lymphoid progenitors (CLPs), early B cells, both immature and mature T cells with notable exceptions, natural killer (NK) and innate lymphoid cell (ILC) populations, fetal macrophages and lymphatic endothelial cells [5]. Transcriptional control is complex, with both positive and negative regulators identified in particular cell populations. For example, the transcription factor NOTCH1 has been shown to be important for initiating IL-7Rα expression in early T cells [31] and SPI1 and, similarly, in early B cells [32], whereas GABP contributes to expression in both lineages [32,33]. Conversely, downregulation of expression is mediated by GFI1 in CD8+ T cells [34] and FOXP3 in regulatory T (Treg) cells [35].

IL-7Rα has been shown to be critical for early T cell-lineage-progenitor generation and expansion [36], including the development of so-called double-negative (DN) early T cells, is indispensable for the transition from the DN2 to DN3 stage for unconventional γδ T cells [37,38], and also for facilitating the DN3 to DN4 transition for conventional αβ T cells [39]. It is also important for the T cell homeostasis, particularly the long-term maintenance of mature T cells in the periphery, including both naïve and memory T (Tm) cells [40]. IL-7R signaling is also critical for B cell development from CLPs, especially from the pro-B to pre-B stages [41,42], influencing differentiation by activating lineage-specific transcription factors such as EBF [43] as well as impacting Ig rearrangement [44]. IL-7R signaling also contributes to the homeostasis of other immune cell populations, including ILC homeostasis, particularly the development and function of ILC2 and ILC3 cells [45,46,47], and the survival and homeostasis of thymic but not conventional NK cells [48] as well as of specific NK cell subsets, such as the non-cytotoxic cytokine-producing CD56high population [49] and RORγt+ NKT cells in tissues [50]. It further contributes to the development of fetal macrophages, particularly tissue-resident forms [51]. Finally, it has been shown to play an essential role in the lymphatic expansion that underpin the final stages of lymph node formation [52,53]. Levels of IL-7Rα are critical, with overexpression shown to reduce IL-7 availability and thereby impact normal development [54]. Conversely, activated T cells lose IL-7Rα expression and become insensitive to IL-7, with IL-7Rα expression and IL-7 responsiveness being restored in long-term Tm cells [55].

## 3. Disruption of IL-7R Signaling Pathways in Immune Cell Diseases

**Figure 2:**
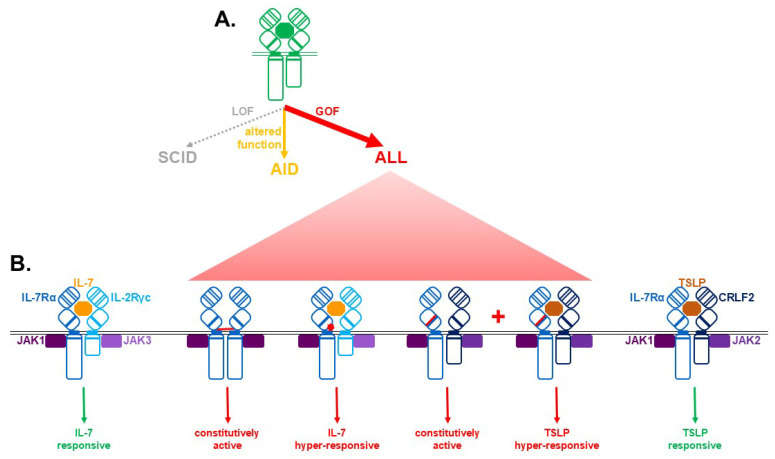
IL-7Rα mutations and disease. (**A**) Overview of different classes of IL-7Rα mutations, including loss-of-function (LOF), resulting in severe combined immunodeficiency (SCID, grey), the altered function associated with susceptibility to autoimmune disease (AID, orange) or gain-of-function (GOF), leading to acute lymphoblastic leukemia (ALL, red). (**B**) Comparison of the ligand-dependent signaling mediated by the wild-type IL-7R (left) and TSLPR (right) complexes with that observed with major classes of mutation found in ALL: extracellular juxtamembrane (EJM) cysteines, leading to constitutive activation of IL-7Rα dimers, EJM positively-charged residues making the IL-7R complex hyper-responsive and CHD S185C, which makes the TSLP receptor constitutively-active and hyper-responsive (middle).

A variety of immune cell diseases are associated with disruption of normal IL-7R signaling, often involving mutations and variants in IL-7R signaling components. These include germline loss-of-function (LOF) mutations associated with immunodeficiencies, more subtle mutations and variants associated with various autoimmune conditions and acquired gain-of-function (GOF) mutations associated with immune cell cancers (Figure 2A).

### 3.1. Severe Combined Immunodeficiency (SCID)

Severe combined immunodeficiency (SCID) represents a suite of primary immune disorders that cause severe impacts on cells within the lymphoid lineage, with significant reductions in specific immune cell populations and/or compromised immune functions, including antibody production. Consequently, patients typically present with recurrent respiratory tract infections, pneumonia, meningitis and failure to thrive, which is associated with early mortality if left untreated [56].

Approximately 10% of human SCID cases are due to LOF mutations in the gene encoding IL7Rα. This autosomal recessive disorder is characterized by so-called T−B+NK+ SCID, in which T cells are specifically ablated, and which is typified by severe infections [57]. The majority of the mutations impact the sequences encoding the IL-7Rα extracellular region or mediate aberrant splicing [58].

Other types of SCID are caused by LOF mutations impacting other IL-7R signaling components, including another autosomal recessive form due to JAK3 mutations [59] and an X-linked form of the disease due to IL2Rγc mutations [60]. Both represent the broader T−B+NK− type of SCID due to their ability to impact the signaling of other IL-2R family members. However, it is generally acknowledged that the effects on T cell numbers are largely the result of disrupted IL-7R signaling.

### 3.2. Autoimmune Disease (AID)

Autoimmune disease (AID) refers to a multitude of disorders underpinned by a disruption of immune tolerance leading to inappropriate immune responses to autoantigens, with the resultant inflammation causing pathology of specific tissues and organs [61]. The nature and location of the autoantigen delineate the respective disorders, with common forms being type I diabetes (T1D), rheumatoid arthritis (RA), multiple sclerosis (MS) and systemic lupus erythematosus (SLE).

AIDs are typically polymorphic with significant environmental interaction, but rare primary forms of the disease exist [62]. Most notable are a range of hypomorphic mutations in IL-7R signaling components, including IL-7Rα and IL-2Rγc, that underpin Omenn’s syndrome [63]. Patients with this disease display susceptibility to infectious diseases, but also inflammation, including erythroderma and other inflammation with elevated IgE and eosinophilia triggered by clonal expansion of Th2 cells [63]. STAT5B LOF mutations lead to similar autoimmune manifestations in concert with the mild immunodeficiency characterized by chronic infections, diarrhea and eczema—although in this case postnatal growth defects are also observed due to independent impacts on growth hormone signaling [64,65]. These patients have a decreased number and/or functionality of Treg, γδ T, CD8+ Tm and NK cells, along with B-cell hyperactivity and elevated IgE [65,66,67,68]. In contrast, GOF variants of JAK1 variants are associated with autoimmunity and autism [69], while haploinsufficiency of the negative regulator SOCS1 leads to early-onset AID [70].

Variants of human IL-7Rα and IL-7 have also been implicated in AID susceptibility. For example, the IL7R SNP rs6897932 C/T Thr66Ile impacts splicing, leading to increased soluble form [71]. This is associated with susceptibility to SLE [72], T1D [73] and MS [71,74], while the IL-7 SNP rs766736182 is associated with asthma [75]. Even in cases that lack IL-7R signaling pathway mutations or variants, IL-7/IL-7R have been shown to promote AID progression [76], probably through promoting self-reactive clones [77]. Thus, IL-7/IL-7R are elevated in rheumatoid arthritis [78], while the soluble form of IL-7Rα is elevated in SLE [79].

### 3.3. Lymphoid Malignancies

Acute lymphoblastic leukemia (ALL) represents the most common childhood cancer, and while good treatment options are available, impacts of the disease can persist long-term and relapse is common [80]. A suite of GOF mutations have been described in ALL that affect IL-7R signaling pathway components [81], but even when not mutated this pathway is pivotal in disease etiology [82].

Around 50–80% of T cell ALL (T-ALL) cases express IL-7R, with IL-7 being shown to play an enhancing role in this disease [82]. Indeed, overexpression of IL-7Rα has been demonstrated to be an early initiating event in ALL [83] and is mediated through a number of mechanisms. This includes increased transcription by activating mutations in the transcription factors NOTCH1 [15] and ZEB2 [84], enhanced expression of the IL-7Rα protein caused by ribosomal-protein L10 mutation [85] and impaired endocytosis of IL-7Rα by mutations in the endocytic regulator dynamin 2 [86].

However, a variety of GOF IL-7Rα mutations also play a key role in ALL (Figure 2B) [4,87]. The most common lesions are insertions and missense mutations that generate unpaired cysteine residues in the extracellular juxtamembrane (EJM) region. This enables IL-7Rα homodimer formation, resulting in constitutive downstream signaling independent of IL-7 or IL-2Rγc. More rarely, such mutations result in positively charged amino acids in the EJM, which still require both IL-7 and IL-2Rγc, but show hypersensitivity to IL-7. In contrast, an S185C mutation in the CHD requires the CRLF2 chain but leads to IL-7-independence and hyperresponsiveness to TSLP. Other mutations lead to a mix of constitutive activation and hypersensitivity [4,87]. GOF IL-7Rα mutations have been found in all forms of T-ALL, but particularly frequently in early T-cell precursor (ETP)-ALL, with insertional EJM mutations predominating [88,89,90]. GOF IL-7Rα mutations have been identified in 2–3% B-ALL [89], mainly resulting from missense mutations, with the S185C CHD mutation being unique to this cohort [87,89]. The incidence of GOF IL-7Rα mutations is considerably higher in so-called Philadelphia (Ph)-like or B cell-precursor (BCP)-ALL, where it is associated with a poor prognosis [87,91].

Amongst other IL-7R signaling pathway components, GOF mutations in JAK1 and JAK3 are commonly observed in T-ALL [92,93], but rarely in B-ALL [93], suggesting they may act through the classic IL-7Rα/IL-2Rγc complex. These mutations are also mutually exclusive with one another and with those in STAT5B and AKT/PTEN in T-ALL, further indicating they all lie in the same pathway [94], with STAT5B mutations being identified as an early event in this disease [95]. Interestingly, GOF IL-7Rα mutations were shown to co-operate with GOF mutations in JAK1 but not JAK3 in an in vitro cell transformation assay [96]. In contrast, GOF JAK2 mutations and translocations are seen in Ph-like/BCP-ALL [97,98], suggesting the IL-7Rα/CRLF2 complex is likely more critical. Indeed, CRLF2 rearrangement and/or overexpression has been observed in around 50% of Ph-like ALL [99].

IL-7Rα mutations appear insufficient to cause leukemia, as has been identified in murine transplantation models [81], and must collaborate with other genes. In pediatric T-ALL and ETP-ALL, concurrent activating mutations in IL-7Rα signaling components and the NRAS/KRAS/NF1 pathway were observed [90,100]. Transduction of primary murine thymocytes with a combination of IL-7R and NRAS mutants resulted in T-ALL in immunocompromised mice [101]. This has been found to be due to augmented MYC activation in concert with elevated BCL2 [102], which is consistent with a zebrafish model showing collaboration between GOF IL-7Rα and MYC in T-ALL [103]. However, other research identified a negative correlation with RAS activating mutations in adult T-ALL [104]. Mutations leading to enhanced expression of HOX genes, especially HOXA and TLX genes, concurrent with GOF IL-7R pathway mutations have been identified in pediatric T-ALL [88,105]. Intriguingly, combining HOXA overexpression with GOF IL-7Rα mutations induced a myeloid malignancy in mice [101], although HOXA9 overexpression was shown to co-operate with GOF JAK3 to drive lymphoid-skewed leukemia [106]. In adult T-ALL, mutations in members of the NOTCH pathway were found to be associated with GOF IL-7Rα mutations [105], with expression of GOF IL-7Rα hastening leukemia development cause by NOTCH1 mutation [107]. Across T-ALL, CDKN2A/B deletion [90] and mutation of epigenetic regulators, such as PHF6, WT1 and PRC, have also been found to associate with IL-7R pathway mutations [105,108]. In BCP-ALL, IL-7R pathway mutations occur concurrently with inactivation of IKZF1 and CDKN2A/B [97]. Silencing of CDKN2A in concert with enforced expression of a GOF IL-7Rα mutant in human CD34+ HSPC enabled the development of BCP-ALL [109], while IKZF1 mutation increased disease penetrance [110].

## 4. IL-7R Pathway-Based Therapies

### 4.1. IL-7

The central role of IL-7R in lymphoid cell biology has seen its ligand IL-7 employed as a treatment strategy in various disease contexts, particularly cancer immunotherapy [111]. For example, IL-7 was shown to enhance immune responses in the context of patient-derived dendritic cell (DC) treatment of metastatic hormone-refractory prostate cancer, with increased expansion of CD4+ and CD8+ T cells and CD56bright NK cells [112]. However, its short half-life has contributed to low efficacy, while higher doses can lead to cytotoxicity [113]. Some of these issues have been addressed, with recombinant forms engineered to incorporate an Fc fragment. One of these, IL-7-Fc, was investigated in the context of CD8+ T cell adoptive cell transfer in a mouse model of melanoma. This molecule enhanced efficacy in a lymphopenic context by increasing the number of adoptively transferred cells with increased tumor inhibition [114]. Interestingly, in an immunocompetent context, it led to preferential expansion of endogenous T cells, resulting in adverse outcomes [114]. An alternate form, NT-17, has been used to manage severe lymphopenia following radiation therapy. In a mouse glioma model treated with radiation and temozolomide, NT-17 increased T lymphocytes, including Tm populations, and enhanced IFNγ production concomitant with increased survival [115]. This treatment has since been subjected to Phase I trials in high-grade glioma patients, where it was well tolerated and increased lymphocyte counts, including CD4+ T cells [116]. As an alternative approach, enforced expression of IL-7 within the immunotherapeutic cell population is proving particularly effective in combination with chemokines in the treatment of solid tumors. Thus, co-expression of IL-7 and CCL-2 has been demonstrated to augment outcomes in the application of chimeric antigen receptor T-cell (CAR-T) treatment of solid tumors [117,118,119] and tumor-antigen-specific TCR T cells [120], with similar positive responses with IL-7 and CCL19 in CAR-T [120,121] and TCR [122] settings. An important caveat of these approaches is the potential for pro-tumor responses, such as enhanced cancer cell invasiveness as observed in prostate cancer cells [123]. IL-7 therapy is also being utilized in the context of viral infections and sepsis [4].

### 4.2. IL-7R

Enforced expression of IL-7Rα has also been applied to augmenting immunotherapy. This includes expression of a CD34-IL-7Rα fusion protein incorporating a constitutively-activating mutation in CAR-T cells, resulted in increased proliferation, activation and cytotoxicity in vitro, with increased CAR-T survival and significant anti-tumor activity in a mouse xenograft model of triple-negative breast cancer [124]. Another approach has been to express a TGFβR/IL-7R fusion in CAR-T cells, thereby converting an immunosuppressive signal to an activating one, which has proven efficacious in a mouse B cell lymphoma model [125].

Treatment of SCID also relies on cell-based therapies, particularly HSC transplantation [58]. However, gene correction approaches are becoming more widely implemented. For example, expression of IL-7Rα using a self-inactivating alpha-retroviral vector has been successfully trialed in a mouse model of IL-7Rα-deficient SICD [126]. However, concerns remain about the potential for malignancy through such approaches [127]. Alternatively, CRISPR/Cas9-mediated genome engineering has been used to insert a corrective gene cassette into the endogenous IL-7Rα locus to restore functionality [128].

### 4.3. Anti-IL-7Rα

For the treatment of leukemias, traditional chemotherapy with or without HSC transplantation remains commonplace [129], although increasingly patient-specific targeting approaches are being used. This includes adult T-ALL patients with IL-7R pathway mutations, with this cohort being shown to be slow responders with allogeneic stem-cell transplantation ineffective [104]. In this regard, anti-IL-7Rα MAb therapy has proven efficacious in ALL [130], with anti-IL-7Rα-drug conjugate being more effective, including in the context of steroid resistance [131]. Anti-IL-7Rα MAb therapy is also being used in the treatment of a range of AIDs, including autoimmune arthritis [132] and T1D [133]. This approach is being extended to the use of conjugates with chemotherapeutic agents, which was shown to be more effective in reducing inflammation in a mouse autoimmune arthritis model [131]. Targeting of IL-7Rα is associated with ablation of vaccine efficacy and anti-viral responses [134], which limit its usefulness in certain patient populations.

### 4.4. IL-7R Pathway Inhibitors

Therapeutic targeting of specific IL-7R downstream signaling components is increasingly being investigated. These include JAK inhibitors, including the JAK1/JAK2 selective Ruxolitinib and Baricitinib, JAK2 (and FLT3) selective Fedratinib and pan-JAK Tofacitinib [135,136]. Amongst these, Baricitinib was successfully used (as part of cocktail of agents) in T-ALL with concurrent JAK1/JAK3/STAT5B mutations [137]. In the case of autoimmune disease, Baricitinib was successful in the context of autoimmune arthritis [138], including in the context of a GOF JAK1 patient [139], while Ruxolitinib was effective in patients with SOCS1 haploinsufficiency [70]. JAK inhibitors can, however, suffer from relatively poor efficacy as well as having potential adverse effects, including through their effects on normal blood and immune cell production and function [135]. Targeting of other pathway components has also produced some promising results. In the context of ALL, this includes mTOR inhibitors that target the PI3K pathway and MEK1/2 inhibitors that target the RAS/MAPK pathway, and which synergize with one another [94] and, potentially, JAK inhibitors [140]. Targeting of BCL family members has also been demonstrated to be effective against ALL [141].

## 5. Future Directions

There remains considerable research to be done in this area. Key amongst this is investigating the role of specific IL-7R pathway mutations and co-operating gene mutations in disease etiology, as well as their differential targeting therapeutically. There is also a need to continue the development of current and novel therapies based on this pathway to increase their effectiveness and scope and/or overcome their deficiencies. Relevant animal models will be essential, including mice, which have already proven critical for understanding the impacts of IL-7R signaling component mutations and their co-operation with other genes [101,102], as well as for evaluating new therapeutics [94,141]. Zebrafish will continue to be a useful adjunct, having shown successful application for examining GOF IL-7Rα mutations and their cooperation with MYC [103] and GOF JAK1 mutations [142], as well as GOF JAK3 mutations and their receptor component requirements and JAK inhibitor sensitivity [143,144]. However, verification and further development of therapeutics in appropriate clinical contexts will be critical in eliciting maximal impact at the individual patient level.

## 6. Conclusions

Cytokine receptor signaling is essential for many aspects of immunology, with specific cytokine receptor chains combining in conjunction with particular downstream JAKs, STATs and other signaling molecules to provide exquisite control. IL-7 signaling via a receptor complex comprising IL-7Rα and IL-2Rγc is part of this sophisticated web. It utilizes JAK1 and JAK3 to mediate signals, particularly via STAT5 and PI3K, to influence lymphoid progenitors, early B and T cells, mature T cells, macrophages and lymphatic endothelial cells. An important aspect of cytokine signaling is its sharply tuned nature, which is such that the loss, gain or other alterations in function can disrupt the balance toward disease. Signaling through the IL-7R pathway exemplifies such a Goldilocks’ equilibrium, with appropriate levels facilitating normal immune development and function, but disturbances cause a range of diseases from SCID through AID to lymphoid malignancies such as ALL. A range of therapeutic approaches are being developed to return affected patients to health, where typically they aim to restore the equilibrium, such as therapeutics targeting IL-7Rα, JAKs and downstream effectors in AID and ALLs. Meanwhile, enhancement of IL-7/IL-7Rα is finding application in cancer immunotherapy.

## Data Availability

Not applicable.

## Abbreviations

The following abbreviations are used in this manuscript:

AID	autoimmune disease
ALL	acute lymphoblastic leukemia
CAR-T	chimeric antigen receptor T cell
CHD	cytokine receptor homology domain
CLP	common lymphoid progenitor
CRLF2	cytokine receptor-like factor 2
EJM	extracellular juxtamembrane
ETP	early T-cell precursor
FNIII	fibronectin type III
FRC	fibroblast reticular cell
GOF	gain-of-function
Ig	immunoglobulin
IL-2Rγc	interleukin-2 receptor gamma common
IL-7R	interleukin-7 receptor
ILC	innate lymphoid cell
JAK	Janus kinase
LOF	loss-of-function
MAb	monoclonal antibody
MS	multiple sclerosis
NK	natural killer
PI3K	phosphatidylinositide 3-kinase
RA	rheumatoid arthritis
SCID	severe combined immunodeficiency
SLE	systemic lupus erythematosus
SOCS	suppressor of cytokine signaling
STAT	signal transducer and activator of transcription
T1D	type 1 diabetes
TCR	T-cell receptor
TEC	thymic epithelial cell
Tm	memory T cell
Treg	regulatory T cell
TSLP	thymic stromal lymphopoietin
